# A Novel circRERE/miR-27a-3p/Caspase9 Signaling Axis Promotes Cardiomyocyte Apoptosis in Ischemic Myocardium: Insights from Epigallocatechin Gallate-Primed Exosomes

**DOI:** 10.3390/cells15090757

**Published:** 2026-04-23

**Authors:** Haiqi Li, Maoqin Wang, Yuxue Li, Xiaowen Gan, Ronggan Liang, Jun Lu, Jie Jian

**Affiliations:** Guangxi Key Laboratory of Drug Discovery and Optimization, Guilin Medical University, Guilin 541199, China

**Keywords:** acute myocardial infarction, circRERE, cardiomyocyte apoptosis, exosomes, EGCG, miR-27a-3p, Caspase9

## Abstract

**Highlights:**

**What are the main findings?**

- circRERE is a novel target for the treatment of acute myocardial infarction.
- Epigallocatechin gallate-primed exosomes alleviated AMI injury via the circRERE/miR-27a-3p/Caspase9 axis.

**What are the implications of the main findings?**

- circRERE is expected to serve as a novel and effective biomarker and therapeutic target for the diagnosis and treatment of myocardial ischemic injury.
- Epigallocatechin gallate-primed exosomes provide a novel natural delivery strategy to treat ischemic heart disease.

**Abstract:**

Acute myocardial infarction (AMI) causes high mortality, with cardiomyocyte apoptosis playing a critical role. Although circular RNAs modulate cardiac disorders, related mechanisms remain unclear. Here, we identify circRERE as a previously unrecognized pro-apoptotic regulator under ischemic stress. circRERE is markedly upregulated in ischemic myocardium and promotes apoptosis by sponging miR-27a-3p to elevate Caspase9. Using epigallocatechin gallate-primed exosomes (EGCG-primed exosomes, Exo^EGCG^) as a tool to modulate circRERE, we found that Exo^EGCG^ significantly reduced circRERE levels, restored miR-27a-3p activity, and suppressed Caspase9. Gain- and loss-of-function tests confirmed that circRERE mediates Exo^EGCG^-derived protection. Collectively, circRERE represents a novel and actionable target for AMI, with Exo^EGCG^ serving as an effective delivery platform.

## 1. Introduction

Acute myocardial infarction (AMI) remains a leading cause of global mortality [1]. Myocardial apoptosis is a critical pathological process contributing to AMI-related cardiac damage, and its inhibition represents a promising strategy for improving functional recovery [2,3]. However, limitations in our understanding of the underlying molecular determinants constrain the development of effective anti-apoptotic therapies.

Circular RNAs (circRNAs), derived from eukaryotic protein-coding genes and predominantly localized in the cytoplasm, exhibit greater stability than their linear counterparts [4,5]. Evidence indicates that cardiomyocyte-expressed circRNAs can modulate cellular apoptosis, often through interactions with microRNAs (miRNAs) [4,5]. Despite this, the specific pathophysiological role of circRERE in AMI, as well as the potential functional crosstalk between circRNAs and Caspase signaling pathways during AMI, remains largely unexplored.

Epigallocatechin gallate (EGCG) exhibits potent cardioprotective effects, but its clinical application is limited by poor bioavailability and instability [6,7]. Developing advanced delivery systems or exploiting its active derivatives to enhance its circulation stability and targeted accumulation is essential for improving therapeutic efficacy [8,9].

Exosomes are extracellular vesicles (30–150 nm in diameter) that carry diverse cargo, including proteins, lipids and nucleic acids, facilitating intercellular communication [10]. They perform a vital role in numerous physiological and pathological processes, serving as natural nanoplatforms for bioactive molecule delivery [10,11]. Critically, recent research substantiates that pretreatment with chemical compounds can modify exosomal content and function, offering significant potential for exosome engineering and therapeutic development [12,13]. We also reported that EGCG enhances exosome secretion from hypoxic cardiomyocytes [13]. However, the functional significance and precise mechanisms of EGCG-induced exosomes (Exo^EGCG^) require further elucidation.

In this study, we first employed bioinformatics tools to identify circRNAs with putative regulatory functions in AMI. Subsequently, leveraging the circRNA/miRNA/mRNA axis framework, we aimed to delineate the therapeutic effects of Exo^EGCG^ and clarify its underlying molecular mechanisms.

Importantly, our circRNA selection criteria prioritized conservation between humans and mice, alongside robust expression and junction ratios in the human heart. This ensured that candidate circRNAs possessed fundamental characteristics conducive to future clinical therapeutic translation. Our findings are anticipated to provide innovative therapeutic strategies for AMI.

## 2. Materials and Methods

### 2.1. Chemicals and Reagents

EGCG (E4143, CAS No.989-51-5, purity ≥95%) and captopril (C4042, CAS No. 62571-86-2, purity ≥98%) were obtained from Sigma-Aldrich Inc. (St. Louis, MO, USA). ExoEasy Maxi Kit (76064) was acquired from QIAGEN (Hilden, Germany). CCK-8 (BS350C) was obtained from Biosharp Biotechnology Co., Ltd. (Guangzhou, China). Invitrogen Lipofectamine 3000 reagent (L3000015) was acquired from Life Technologies (Carlsbad, CA, USA). TUNEL staining kit (E-CK-A320) and cTn-I ELISA kit (E-EL-M1203) were obtained from Elabscience Biotechnology Co., Ltd. (Wuhan, China). Trizol reagent (DP424) was obtained from Tiangen Biotechnology Co., Ltd. (Beijing, China). Primary antibodies against Caspase9 (ab202068), cleaved Caspase3 (ab184787), and GAPDH (ab9485) were acquired from Abcam (Cambridge, UK). Reverse transcription system (AT311, AT351) and QPCR Master Mix (AQ601) were purchased from Trans Gen Biotech Biosharp Biotechnology Co., Ltd. (Beijing, China). RIP kit (Bes5101) was obtained from BersinBio Co., Ltd. (Guangzhou, China).

### 2.2. Cell Culture and Hypoxia Model Establishment

HL-1 cardiomyocytes or 293T cells were obtained from iCell Bioscience Inc. (Shanghai, China) or the National Collection of Authenticated Cell Cultures (Shanghai, China). Prior to hypoxia induction, HL-1 cells were co-cultured with the relevant exosomes for 24 h, according to the experimental design (Table S1). Subsequently, the cells were maintained in serum-free medium under hypoxic conditions (anaerobic incubator) for 12 h.

### 2.3. Exosome Extraction and Identification

Exosomes were isolated from the culture supernatants of hypoxic HL-1 cardiomyocytes, either pretreated with EGCG or not (recorded as Exo^EGCG^ or Exo^Hypoxia^, respectively), using the exoEasy Maxi Kit. Exosomes were characterized by Western blotting, nanoparticle tracking analysis (NTA, Malvern Instruments, Malvern, UK) and transmission electron microscope (TEM, HITACHI, HT7700, Tokyo, Japan). To assess exosome uptake, exosomes were labeled with PKH67 or DiR in vitro or in vivo.

### 2.4. Transient Transfection

All plasmids were constructed by Jikai Gene Technology (Shanghai, China). Plasmid or corresponding NC was transfected according to the instructions of Invitrogen Lipofectamine 3000.

### 2.5. Animal Treatment and AMI Model Establishment

The animal procedures were conducted in compliance with the Guide for the Care and Use of Laboratory Animals and approved by the Animal Ethics Committee of Guilin Medical University (GLMC 202003304). Male C57BL/6 mice (22–25 g) were randomly assigned to experimental groups. Based on the study design, Exo^EGCG^, si-circRERE, antagomiR-27a-3p or their corresponding negative control (NC) was administered prior to ischemia (Table S2). The AMI model was induced by ligating the left anterior descending coronary artery for 12 h. While the AMI model was established, the success criteria were determined by ST-segment elevation, tall or inverted T waves in lead II electrocardiogram manifestations. After the ischemic period, mice were anesthetized with isoflurane, after which myocardial tissues and serum were collected and stored at −80 °C for subsequent analysis.

### 2.6. Cell Viability Assay

CCK-8 assay kit was used following instruction.

### 2.7. ELISA

CTn-I levels were measured according to the technical manual.

### 2.8. TUNEL Staining

The apoptosis rate of myocardia or cardiomyocytes was detected by TUNEL staining based on the experiment protocol.

### 2.9. RT-qPCR Analysis

Total RNA was isolated using Trizol reagent. cDNA was produced with transcript cDNA synthesis system and then amplified using QPCR Master Mix. All procedures were implemented strictly following manufacturers’ manuals. For mRNA or miRNA quantification, *GAPDH* or U6 was performed as internal reference gene, respectively. Data analysis was conducted via 2^−ΔΔCt^ method. Primer sequences are listed in Table S3.

### 2.10. Western Blotting Analysis

Protein extracts from cardiomyocytes or myocardium were separated by SDS-PAGE, then transferred to a membrane, and blocked. The membrane was then incubated sequentially with primary and secondary antibodies. Protein bands were observed and documented with gel imaging system (Syngene, Cambridge, UK), then band intensity was quantitated through ImageJ 1.8.0 software (Rawak Software Inc., Germany).

### 2.11. Echocardiography Detection

After 12 h of ischemia, mice were anesthetized with isoflurane. Left ventricular ejection fraction (EF) and fractional shortening (FS) were automatically calculated from three consecutive cardiac cycles to evaluated cardiac function with echocardiography (VINNO X6, Suzhou, China).

### 2.12. circRERE Circularization and Cell Localization Detection

Convergent primer and divergent primer of circRERE were designed to amplify genomic DNA (gDNA) and complementary DNA (cDNA), then gel electrophoresis and Sanger sequencing were implemented. To conduct circRERE’s subcellular localization, cytoplasmic and nuclear RNA fractions were isolated from HL-1 cells. RT-qPCR was then executed to assess the relative enrichment of circRERE in each compartment.

### 2.13. Dual-Luciferase Reporter Assay

Respective wild-type (WT) and mutant (MUT) reporter vector plasmids were constructed by Sangon Biotech (Shanghai, China). The miR-27a-3p mimic or NC was co-transfected with the WT or MUT into 293T cells. The dual-luciferase activity was then measured following the manufacturer’s instructions.

### 2.14. RNA Immunoprecipitation (RIP) Test

Following transfection with either miR-27a-3p mimic or NC plasmid, immunoprecipitation was performed. RNA was extracted according to the manual’s instructions. Then, the binding efficiency of circRERE was detected by RT-qPCR.

### 2.15. Bioinformatics Analyses

By analyzing the sequencing data of the GSE24548 dataset in the GEO database, differentially expressed miRNAs in AMI were identified [14]. To identify circRNAs interacting with the objective miRNA, we queried the ENCORI database, extracting the number of supporting AGO CLIP-seq experiments (AgoExpNum) for each interaction [15]. Subsequently, the circBase database was utilized to filter for circRNAs exhibiting high sequence conservation between humans and mice [16]. Finally, the circAtlas database was employed to obtain the expression levels (circExp) and junction ratios (JncRto) of these conserved circRNAs across various human tissues [17].

### 2.16. Statistical Analyses

Data were presented as means±SD. Comparison between two groups was tested by t-test. Variations among multiple groups were tested using one-way ANOVA after confirming normality and homogeneity of variances, followed by Tukey’s post hoc comparisons. *p <* 0.05 was considered as statistical significance. All calculations were performed in GraphPad Prism 5 (San Diego, CA, USA).

## 3. Results

### 3.1. Characterization of Exosomes

**Figure 1:**
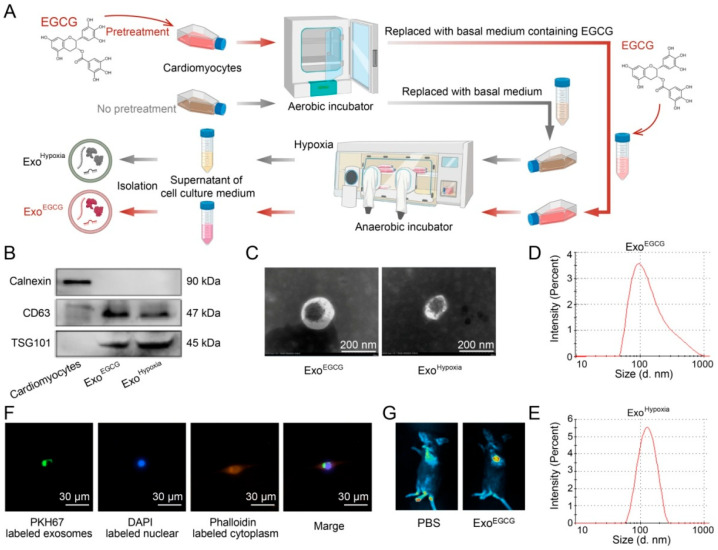
Characterization and uptake of Exo^EGCG^. (**A**) Schematic presentation showing the preparation process of Exo^EGCG^ and Exo^Hypoxia^. (**B**) Exo^EGCG^ and Exo^Hypoxia^ expressed exosomal protein markers (CD63, TSG101), but not the tissue-specific protein (calnexin). (**C**) Cup-shaped morphologies of Exo^EGCG^ and Exo^Hypoxia^ were assessed by TEM. Scale bars, 200 nm. The particle sizes of (**D**) Exo^EGCG^ and (**E**) Exo^Hypoxia^ were 100–150 nm. Exosomes were internalized by (**F**) cardiomyocytes and (**G**) myocardium. Scale bars, 30 μm.

Exosomes were isolated from EGCG-treated cardiomyocytes (Figure 1A). These extracellular vesicles exhibited the exosomal markers CD63 and TSG101, while calnexin, a tissue-specific protein, was absent (Figure 1B). Furthermore, the vesicles displayed typical exosomal morphology and phenotype, confirming successful isolation and purification (Figure 1C–E). Importantly, PKH67- or DiR-labeled exosomes were efficiently taken up by HL-1 cardiomyocytes or myocardium (Figure 1F,G).

### 3.2. Screening, Identification and Localization of circRERE

**Figure 2:**
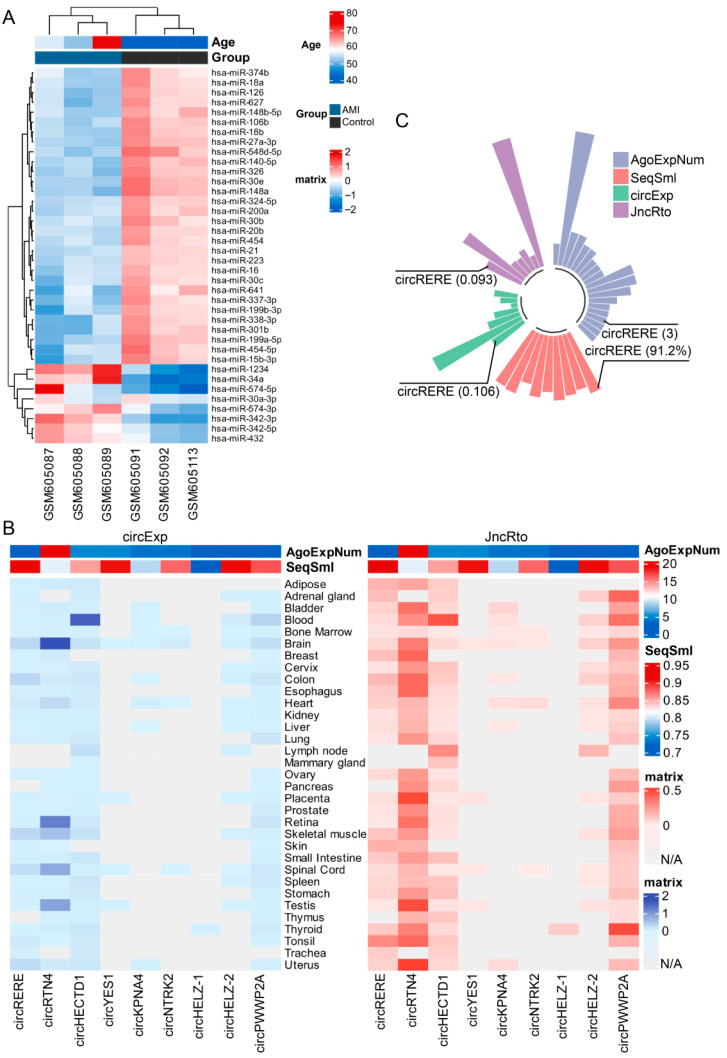
Screening of circRERE and targeting miR-27a-3p. (**A**) Heat map of differentially expressed miRNAs in the serum of AMI patients. (**B**) The number of supported AGO CLIP-seq experiments (AgoExpNum) and sequence similarity (SeqSml) of circRNA interacting with miR-27a-3p, as well as their circRNA expression (circExp) and junction ratio (JncRto) in human organs. (**C**) Values of screening indicators for circRERE.

**Figure 3:**
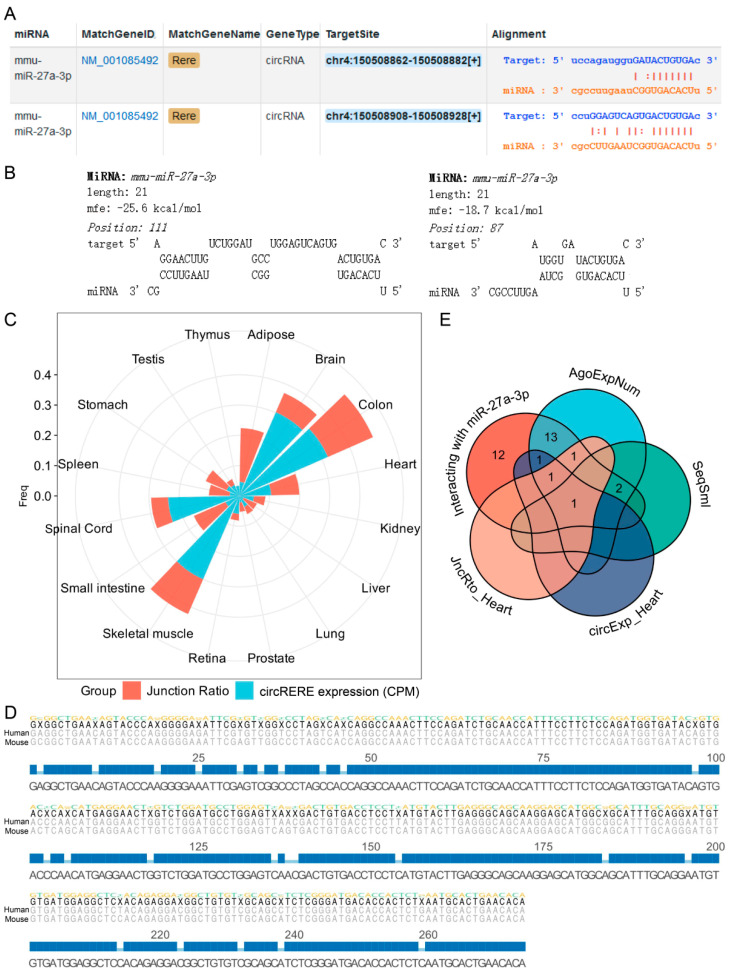
Interaction analysis of circRERE. Prediction results for the interaction between circRERE and miR-27a-3p using (**A**) ENCORI database and (**B**) RNAhybrid software 2.1.2. (**C**) circRERE demonstrated considerable junction ratio and expression in multiple human organs. (**D**) The homology of circRERE between human and mouse was higher than 90%. (**E**) Venn diagram of circRNAs that met the individual screening criteria.

We first identified miR-27a-3p, whose expression was downregulated in AMI patients (Figure 2A), as a candidate miRNA. Subsequently, we retrieved circRNAs that interact with miR-27a-3p from the ENCORI database, and collected the number of supporting AGO CLIP-seq experiments (AgoExpNum), sequence similarity (SeqSml) between humans and mice, circular transcript expression level (circExp) and junction ratio (JncRto) across various human tissues for these circRNAs using the circBase and circAtlas databases (Figure 2B). Notably, among all candidate circRNAs, circRERE exhibited comprehensively superior and well-balanced performance across the four key evaluation metrics mentioned above (Figure 2C). Predictions from the ENCORI and RNAhybrid databases indicated that miR-27a-3p may have a targeted binding relationship with circRERE (Figure 3A,B). Meanwhile, circRERE exhibited not only a high junction ratio but also significantly elevated expression level in human cardiac tissue (Figure 3C). Further sequence alignment results demonstrated that the sequence homology of circRERE between humans and mice exceeded 90% (Figure 3D). Unlike other circRNAs that only showed advantages in individual indicators, no obvious deficiencies were observed in circRERE for any single metric (Figure 3E). This balanced superiority suggested that it was the most reliable candidate for subsequent in vivo and in vitro experiments.

**Figure 4:**
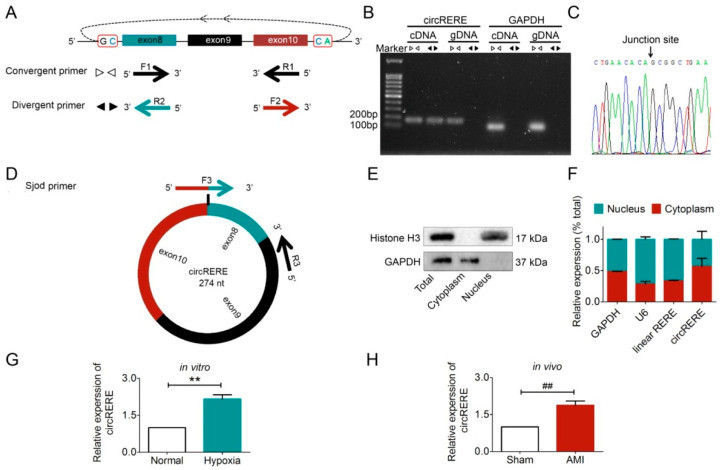
Identification and localization of circRERE. (**A**) Schematic representation of circRERE and its convergent primers and divergent primers. (**B**) The circular structure of circRERE was verified by agarose gel electrophoresis; white triangles refer to convergent primers, while black triangles represent divergent primers. (**C**) The circRERE junction site was validated by Sanger sequencing. (**D**) Schematic representation of circRERE and its splice junction overlapping divergent (Sjod) primers. (**E**) The separation effect of the cytoplasm and the nucleus was good. (**F**) The relative content of circRERE in the cytoplasm was higher. The level of circRERE was upregulated in (**G**) hypoxic cardiomyocytes and (**H**) AMI mice, *n*  =  5. ** p < 0.01 vs. Normal; ^##^ *p* < 0.01 vs. Sham.

Secondly, to confirm the circular structure of circRERE, we designed a convergent primer and a divergent primer (Figure 4A). Using cDNA as a template, both primer sets generated amplification products (Figure 4B). However, only the convergent primer produced an amplification product when genomic DNA (gDNA) was used as a template (Figure 4B), which confirmed the circular structure of circRERE and excluded the possibility of false positives arising from *RERE* gene recombination. Sanger sequencing analysis precisely defined the junction sequence as 5′-ctgaacacaccggctgaa-3′ (Figure 4C). Then, we designed the splice junction overlapping divergent primer (Sjod primer) for exclusive amplification of circRNAs containing the above-mentioned precise junction sequence (Figure 4D).

Thirdly, we demonstrated that circRERE predominantly localizes to the cytoplasm of cardiomyocytes, fulfilling the spatial requirement for miRNA interaction as a ceRNA. Following effective separation of cytoplasmic and nuclear fractions from HL-1 cardiomyocytes (Figure 4E), RNA was extracted. RT-qPCR analysis results confirmed that compared to the nucleus, the level of circRERE in the cytoplasm is significantly higher (Figure 4F).

Finally, we verified that circRERE expression is upregulated during AMI, both in vitro and in vivo (Figure 4G,H).

### 3.3. circRERE Exacerbated AMI

**Figure 5:**
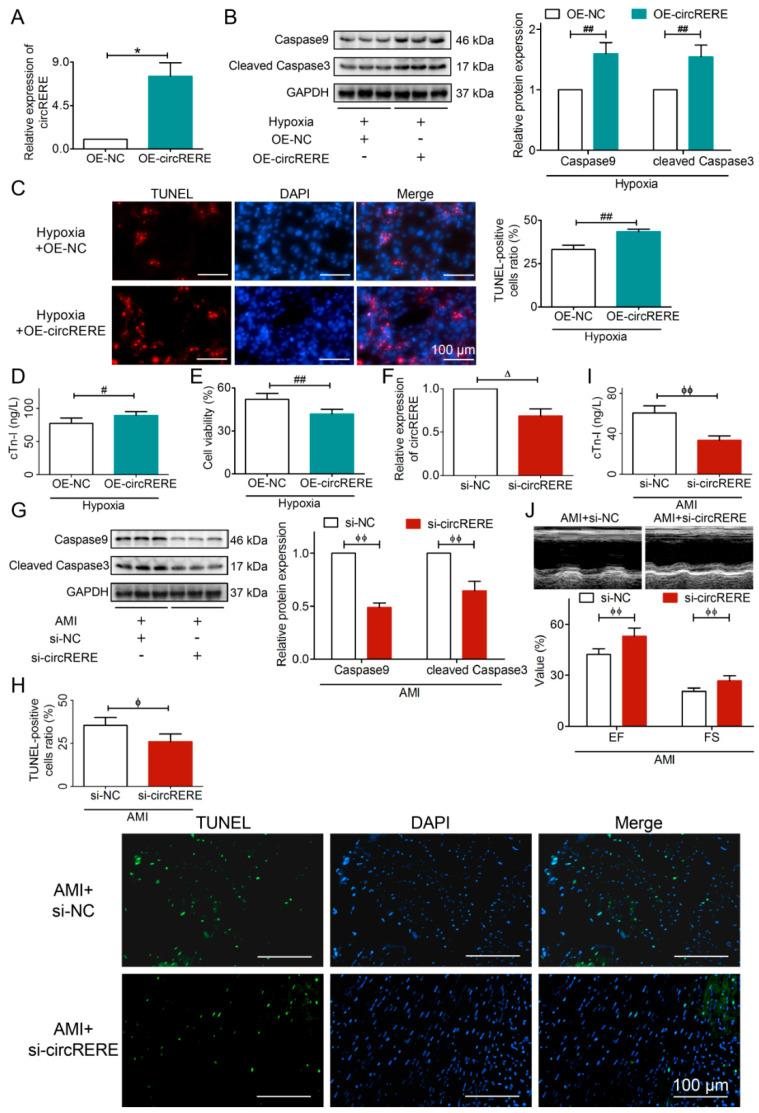
circRERE aggravated AMI injury by exacerbating apoptosis. Transfection effect of (**A**) OE-circRERE and (**F**) si-circRERE, *n*  =  3. Overexpression of circRERE (**B**) upregulated the levels of pro-apoptotic proteins Caspase9 and cleaved Caspase3, (**C**) increased the rate of apoptosis, (**D**) elevated the level of cTn-I, and (**E**) decreased cell viability. Knockdown of circRERE (**G**) downregulated the levels of pro-apoptotic proteins Caspase9 and cleaved Caspase3, (**H**) reduced the rate of apoptosis, *n*  =  3, (**I**) lowered the levels of cTn-I, and (**J**) elevated EF and FS, which improved cardiac function. *n*  =  5; * p < 0.05 vs. OE-NC; ^#^ *p* < 0.05, ^##^ *p* < 0.01 vs. Hypoxia + OE-NC; ^Δ^ *p* < 0.05 vs. si-NC; ^ϕ^ *p* < 0.05, ^ϕϕ^ *p* < 0.01 vs. AMI + si-NC.+, treated; −, not treated. Normal cardiomyocyte viability was considered as 100% viability. Scale bars, 100 μm.

To elucidate the function of circRERE in AMI injury, we performed a gain-of-function experiment. Plasmid overexpressing of circRERE was transfected into hypoxia HL-1 cells (Figure 5A). circRERE overexpression significantly upregulated the apoptosis markers Caspase9 and cleaved Caspase3 (Figure 5B). Additionally, TUNEL, ELISA, and CCK-8 assays confirmed the pro-apoptotic and myocardial injury-promoting effects of circRERE overexpression (Figure 5C–E). Collectively, circRERE exacerbates cellular injury in AMI.

Given the evolutionary conservation of circRERE between humans and mice, we investigated its functional role in myocardial infarction in vivo. For loss-of-function studies, si-circRERE was delivered via myocardial stereotactic injection in AMI mice, with transfection efficiency confirmed by RT-qPCR (Figure 5F). Knockdown of circRERE significantly reduced Caspase9 and cleaved Caspase3 (Figure 5G), decreased apoptosis rates, and lowered cTn-I contents (Figure 5H,I). Moreover, echocardiography showed ameliorated cardiac function, as supported by elevated EF and FS (Figure 5J). In summary, the silencing of circRERE mitigates AMI injury.

Both in vitro and in vivo experiments consistently highlighted circRERE as a potential therapeutic target for AMI.

### 3.4. circRERE Regulated miR-27a-3p as a ceRNA

**Figure 6:**
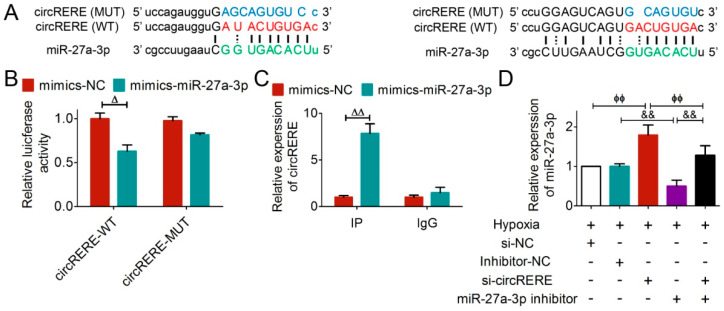
circRERE served as a sponge for miR-27a-3p. (**A**) Schematic diagram of the binding sites for miR-27a-3p and circRERE and the corresponding mutation. (**B**) Dual-luciferase reporter assay and (**C**) RIP assay verified the interaction between circRERE and miR-27a-3p, *n* = 3. (**D**) Silencing of circRERE partially reversed the effect of miR-27a-3p inhibitor, *n*  =  5. ^Δ^ *p* < 0.05, ^ΔΔ^ *p* < 0.01 vs. mimics-NC; ^ϕϕ^ *p* < 0.01 vs. Hypoxia + si-circRERE; ^&&^ *p* < 0.01 vs. Hypoxia + miR-27a-3p inhibitor. +, treated; −, not treated.

To investigate whether miR-27a-3p directly binds to circRERE, we performed a dual-luciferase reporter assay (Figure 6A). Cells carrying the wild-type circRERE (circRERE-WT), when co-transfected with miR-27a-3p mimics, showed notably diminished luciferase activity (Figure 6B), but miR-27a-3p mimics had no effect on the mutant construct (circRERE-MUT) (Figure 6B). Additionally, RIP assays confirmed circRERE enrichment in miR-27a-3p-overexpressing HL-1 cells (Figure 6C), further supporting their interaction under myocardial physiological conditions.

In hypoxic HL-1 cells, miR-27a-3p levels were elevated compared to controls. The silencing of circRERE partially reversed the effects of miR-27a-3p inhibitor (Figure 6D), suggesting a regulatory relationship between them.

All in all, the preceding results validated that circRERE serves as a competitive sponge for miR-27a-3p in AMI.

### 3.5. Knockdown of miR-27a-3p Exacerbated AMI Injury

**Figure 7:**
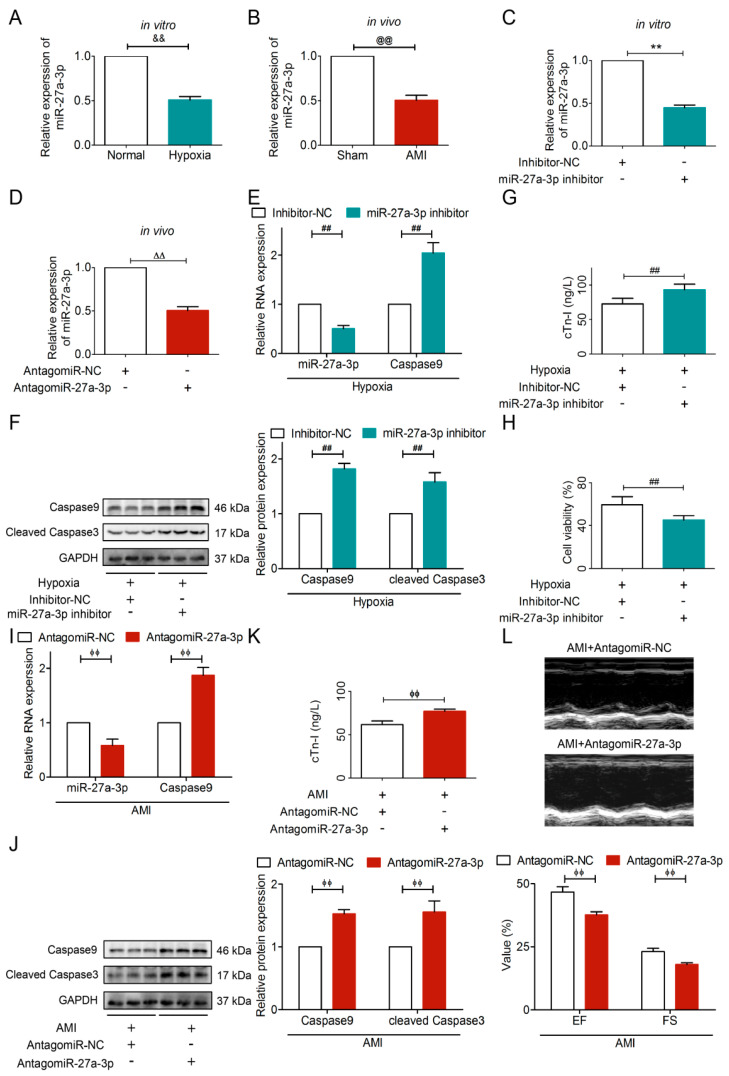
Knockdown of miR-27a-3p exacerbated AMI injury by aggravating apoptosis. The level of miR-27a-3p was downregulated in (**A**) hypoxic cardiomyocytes and (**B**) AMI mice. Transfection effect of (**C**) miR-27a-3p inhibitor and (**D**) antagomiR-27a-3p, *n* =  3. In vitro, knockdown of miR-27a-3p (**E**) decreased miR-27a-3p and increased *Caspase9* RNA expression, (**F**) upregulated protein expression of Caspase9 and cleaved Caspase3, (**G**) increased cTn-I, (**H**) inhibited cell viability. In vivo, downregulation of miR-27a-3p (**I**) decreased miR-27a-3p and increased *Caspase9* RNA expression, (**J**) upregulated protein expression of Caspase9 and cleaved Caspase3, (**K**) increased cTn-I, (**L**) decreased EF and FS. *n*  =  5, or *n*  =  3 which was marked out individually; ^&&^ *p* < 0.01 vs. Normal; ^@@^ *p* < 0.01 vs. Sham; ** *p* < 0.01 vs. Inhibitor-NC; ^∆∆^ *p* < 0.01 vs. AntagomiR-NC; ^##^ *p* < 0.01 vs. Hypoxia + Inhibitor-NC; ^ϕϕ^ *p* < 0.01 vs. AMI + AntagomiR-NC. +, treated; −, not treated. Normal cardiomyocyte viability was considered as 100% viability.

Firstly, we observed the downregulation of miR-27a-3p in ischemia both in vitro and in vivo (Figure 7A,B). Secondly, to knock down miR-27a-3p levels in vitro and in vivo, we designed a miR-27a-3p inhibitor and an antagomiR-27a-3p, respectively (Figure 7C,D). Thirdly, in vitro, transfection of hypoxic HL-1 cardiomyocytes with the miR-27a-3p inhibitor significantly reduced miR-27a-3p concomitantly increased Caspase9, cleaved Caspase3 and the myocardial injury biomarker cTn-I, as well as decreased cell viability (Figure 7E–H). Encouragingly, in vivo experiments yielded consistent results (Figure 7I–L), confirming that miR-27a-3p downregulation promotes apoptosis and exacerbates AMI injury.

### 3.6. Caspase9 Was a Direct Target of miR-27a-3p

**Figure 8:**
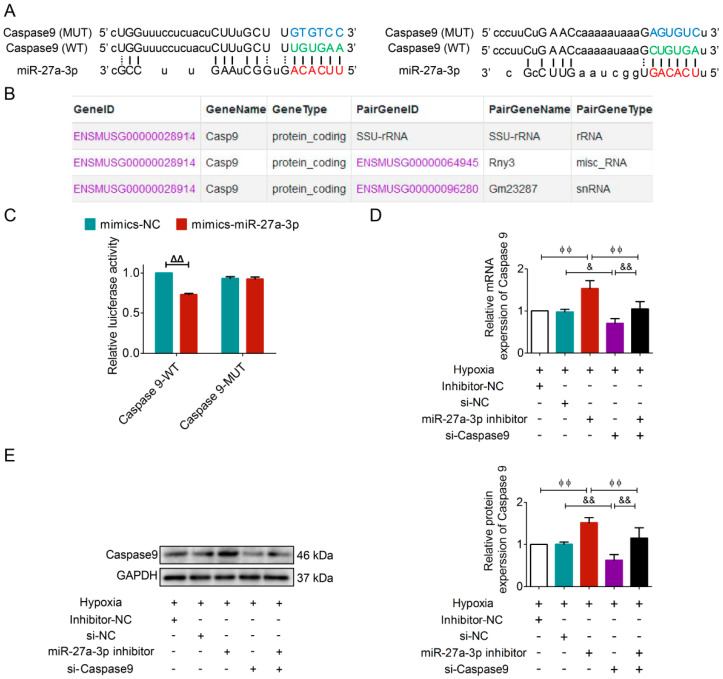
*Caspase9* was a direct target of miR-27a-3p. (**A**) RNAhybrid software was used to predict the potentially binding sites and the corresponding mutation of *Caspase9* and miR-27a-3p. (**B**) ENCORI database predictions showed there is no binding trend of circRERE to *Caspase9*. (**C**) Dual-luciferase reporter assay verified the combination of *Caspase9* and miR-27a-3p, *n*  =  3. (**D**,**E**) miR-27a-3p inhibitor partially reversed the effect of si-Caspase9, *n*  =  5. ^ΔΔ^ *p* < 0.01 vs. mimics-NC; ^ϕϕ^ *p* < 0.01 vs. Hypoxia + miR-27a-3p inhibitor; ^&^ *p* < 0.05, ^&&^ *p* < 0.01 vs. Hypoxia + si-Caspase9. +, treated; −, not treated.

To determine whether a circRNA/miRNA/mRNA axis mediates the regulatory role of circRERE in AMI injury, we used bioinformatics databases, ENCORI and RNAhybrid software 2.1.2 [15,18] to predict potential miRNAs targeting the 3’UTR of *Caspase9*. These analyses identified miR-27a-3p as a candidate miRNA with binding sites on *Caspase9* (Figure 8A). Importantly, circRERE did not directly bind to *Caspase9* (Figure 8B), ruling out a competitive binding mechanism. To validate the interaction, a dual-luciferase reporter assay was implemented in 293T cells. Co-transfection of miR-27a-3p mimic with a wild-type *Caspase9* 3’UTR reporter (WT) significantly reduced luciferase activity, whereas mutation of the predicted miR-27a-3p binding site (MUT) abolished this effect (Figure 8C). Further functional validation in hypoxic cardiomyocytes showed that miR-27a-3p inhibition increased *Caspase9* mRNA and protein levels while partially reversing the effects of *Caspase9* knockdown (Figure 8D,E). The results above validated that miR-27a-3p directly binds to *Caspase9* and suppresses its expression.

### 3.7. Exo^EGCG^ Attenuated AMI Injury by Regulating Apoptosis

Exo^EGCG^ exhibited no obvious toxic effects on cardiomyocytes or myocardium (Figure S1A,B). Exo^EGCG^ exerted dose-dependent cardioprotective effects both in vitro and in vivo, with the most prominent protective efficacy at doses of 50 μg/mL (in vitro) and 2 mg/kg (in vivo) (Figure S1C,D).

Further investigations demonstrated that, compared with the control group, Exo^EGCG^ pretreatment significantly enhanced cardiomyocyte viability, reduced cTn-I level, decreased the apoptotic rate, and effectively improved cardiac function (Figure S2A–C,E–G). In addition, Exo^EGCG^ alleviated AMI injury by downregulating the expression of Caspase9 and cleaved Caspase3 (Figure S2D,H). Moreover, the aforementioned cardioprotective effects of Exo^EGCG^ were significantly superior to those of Exo^Hypoxia^ (Figure S2).

### 3.8. Exo^EGCG^ Attenuated AMI via circRERE/miR-27a-3p/Caspase9 Axis

**Figure 9:**
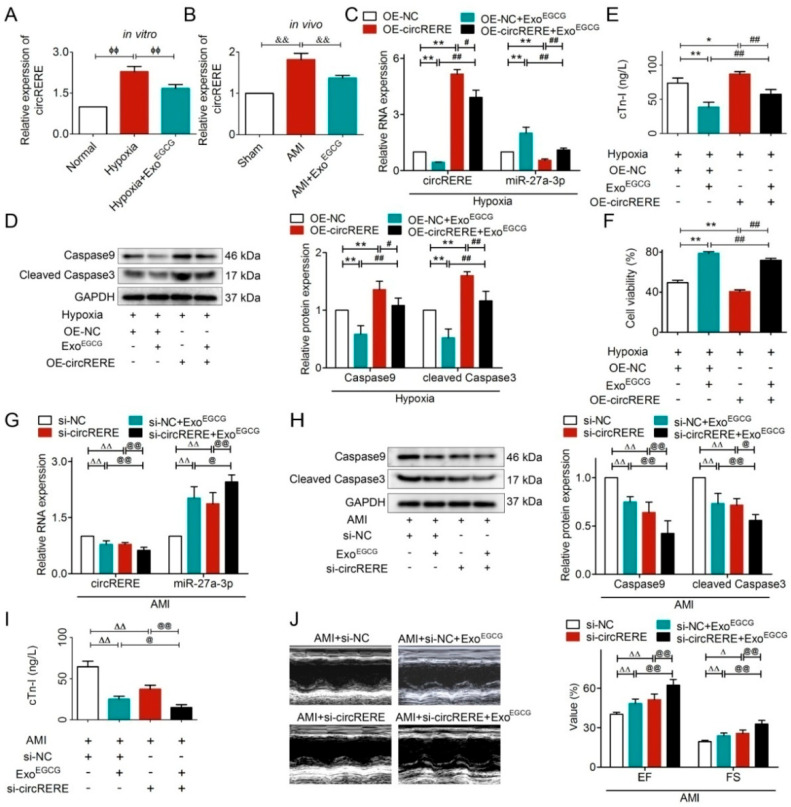
Exo^EGCG^ attenuated AMI by regulating circRERE. The levels of circRERE in AMI were regulated by Exo^EGCG^ (**A**) in vitro and (**B**) in vivo. circRERE overexpression partially reversed the effects of Exo^EGCG^ in (**C**) circRERE and miR-27a-3p levels, (**D**) protein levels of Caspase9 and cleaved Caspase3, (**E**) cTn-I level, (**F**) cell viability, while Exo^EGCG^ also partially reversed the effects of circRERE overexpression mentioned above. Silencing of circRERE synergistically enhanced the effects of Exo^EGCG^ in (**G**) circRERE and miR-27a-3p levels, (**H**) protein levels of Caspase9 and cleaved Caspase3, (**I**) cTn-I level, (**J**) EF and FS, while co-administration of Exo^EGCG^ synergistically enhanced the above-mentioned effects of circRERE silencing. *n*  =  5; ^ϕϕ^ *p* < 0.01 vs. Hypoxia; ^&&^ *p* < 0.01 vs. AMI; * *p* < 0.05, ** p < 0.01 vs. Hypoxia + OE-NC; ^#^ *p* < 0.05, ^##^ *p* < 0.01 vs. Hypoxia + Exo^EGCG^ + OE-circRERE; ^Δ^ *p* < 0.05, ^ΔΔ^ *p* < 0.01 vs. AMI + si-NC; ^@^ *p* < 0.05, ^@@^ *p* < 0.01 vs. AMI + Exo^EGCG^ + si-circRERE. +, treated; −, not treated. Scale bars, 100 μm. Normal cardiomyocyte viability was considered as 100% viability.

Exo^EGCG^ significantly reduced circRERE expression both in vitro and in vivo (Figure 9A,B). Subsequent rescue experiments in vitro revealed that Exo^EGCG^ pretreatment not only downregulated circRERE but also upregulated miR-27a-3p (Figure 9C). Notably, circRERE overexpression partially reversed the effects mentioned above (Figure 9C). Furthermore, circRERE overexpression reversed the effects of Exo^EGCG^, mitigating the reductions in Caspase9, cleaved Caspase3, and cTn-I levels and restoring the diminished cell viability (Figure 9D–F).

To assess the in vivo relevance of circRERE, we performed knockdown experiments in AMI mice. Depleting circRERE synergized with Exo^EGCG^ pretreatment, leading to further suppression of circRERE and an increase in miR-27a-3p expression (Figure 9G). This combined intervention also amplified the cardioprotective effects of Exo^EGCG^, as evidenced by further reduced Caspase9/cleaved Caspase3, lowered cTn-I, and improved cardiac function (Figure 9H–J).

Collectively, the findings above established that circRERE mediated the cardioprotective impacts of Exo^EGCG^. Further gain- and loss-of-function experiments, conducted both in vitro and in vivo, verified the critical role of the circRERE/miR-27a-3p/Caspase9 axis in Exo^EGCG^’s therapeutic mechanism (Figures S3 and S4).

## 4. Discussion

CircRNAs are increasingly acknowledged as pivotal regulators in the modulation of apoptosis and AMI [4,5]. Using the ENCORI database, we first analyzed the differentially expressed miRNAs in AMI patients and initially screened out the candidate circRNAs that may have important functions. Next, to identify a candidate circRNA for further investigation, we evaluated four key metrics: the number of supporting AGO CLIP-seq experiments (AgoExpNum), human–mouse sequence similarity (SeqSml), expression level (circExp), and junction ratio (JncRto) of each circRNA. Specifically, high AgoExpNum indicates that its binding to miRNAs is reproducible and stable, which eliminates accidental experimental errors and significantly enhances the credibility of the circRNA possessing biological functions [15,19,20]. SeqSml reflects the evolutionary conservation and functional necessity of circRNAs. A high sequence similarity of homologous circRNAs between humans and mice suggests that these circRNAs have been retained during mammalian evolution, implying that their functions are indispensable for life activities. Nucleic acid sequences with high evolutionary conservation usually exert critical biological functions, and this indicator helps screen circRNAs with cross-species functional potential, making it more likely for the research conclusions obtained from mouse models to be extrapolated to humans [19,20]. In addition, highly expressed circRNAs enhance the operability and feasibility of downstream experiments. Their robust expression facilitates verification and quantification across diverse experimental approaches, which simplifies subsequent functional studies [17,19,20]. As shown in Figure 2 and Figure 3, the high circExp score of circRERE, in particular, overcomes the common limitation of tissue-specific expression, confirming its stable presence in cardiac tissue. JncRto is a core indicator for judging the actual existence of circRNAs. A high junction ratio demonstrates that the formation of the circular structure of the circRNA is specific and stable, which can effectively exclude false-positive circRNAs caused by RNA degradation and sequencing errors, thus endowing them with substantial research value [17,19,20]. Notably, unlike other candidates that exhibited distinct deficiencies in the above-mentioned metrics, circRERE (circBase ID: mmu_circ_0001305) achieved excellent performance in every single dimension without any critical weaknesses that might compromise subsequent experiments. This well-defined expression pattern makes it an ideal candidate for investigating the functional roles and regulatory networks of circRNAs in AMI.

We first investigated the expression pattern and function of circRERE in AMI. Experimental results showed that the expression level of circRERE was significantly upregulated (Figure 4G,H). Given that circRNAs typically exert their miRNA-sponge functions in the cytoplasm [5,21], we then verified the subcellular localization of circRERE. As shown in Figure 4, circRERE was predominantly cytoplasmic, fulfilling the spatial requirement. With the help of RNAhybrid software and the ENCORI database [15,18], we predicted that miR-27a-3p and circRERE might have interactions. Subsequent luciferasereporter assay and RIP test validated that circRERE acts as a competitive endogenous RNA (ceRNA). We further discovered that miR-27a-3p and circRERE had direct interactions in mouse cardiomyocytes.

To better understand the role of circRERE on AMI, we constructed a plasmid for circRERE overexpression in vitro and a siRNA for circRERE silencing in vivo. Subsequent gain-of-function and loss-of-function experiments revealed that circRERE could suppress cell viability, aggravate the myocardial injury, and promote cell apoptosis by upregulating Caspase9 and cleaved Caspase3. Additionally, knockdown of circRERE upregulated miR-27a-3p, which in turn suppressed Caspase9 expression. These findings suggested that circRERE and miR-27a-3p may serve as upstream regulators of Caspase9-mediated apoptosis. Accumulatively, our studies established that circRERE acts as a molecular sponge for miR-27a-3p, thus promoting apoptosis in ischemic/hypoxic cardiomyocytes. For the first time, we clearly elucidated the unreported pathophysiological function of circRERE in AMI and identified a regulatory axis involving circRERE, miR-27a-3p, and Caspase9. Notably, circRERE shows remarkable cross-species conservation, with >90% sequence homology between humans and mice, which strongly highlights its translational relevance.

Cardiomyocytes, although not conventional secretory cells, could release exosomes under stressful conditions such as hypoxia and ischemia [13]. Notably, external stimuli, including pharmacological agents and alterations in the cellular microenvironment, can significantly modulate exosome biogenesis and cellular uptake [12,13,22,23]. Therefore, investigating natural bioactive compounds that modulate exosome-mediated cardiac communication offers a promising avenue for developing novel multi-target therapies against AMI.

EGCG, the bioactive polyphenol in green tea, exhibits potent antioxidant activity [7]. Current research primarily emphasizes EGCG’s applications in oncology, while evidence remains scarce regarding its cardioprotective effects mediated by non-coding RNA regulation, particularly via the exosomal pathway [6,7]. As shown in Figure S2, compared to Exo^Hypoxia^, Exo^EGCG^ markedly increased cell viability and reduced cTn-I level, indicating that EGCG markedly improves the cardioprotective efficacy of cardiomyocyte-secreted exosomes. Further mechanistic studies confirmed that Exo^EGCG^ exerts opposing biological effects to circRERE in AMI. Then, we investigated whether Exo^EGCG^ could regulate the functions of circRERE in AMI. As shown in Figure 9A,B, Exo^EGCG^ downregulated the expression level of circRERE. Further mechanistic studies confirmed that Exo^EGCG^ attenuated AMI injury via the circRERE/miR-27a-3p/Caspase9 regulatory axis.

Our research proposes a novel strategy to improve the therapeutic efficacy of EGCG using an exosome-based nanodelivery platform. As established in the literature, exosomes offer distinct advantages as natural nanocarriers, including enhanced cellular uptake, excellent biocompatibility, and greater stability in vivo [10,11]. Their lipid bilayer membrane provides a protective barrier that shields encapsulated therapeutics—such as nucleic acids and small molecules—from enzymatic degradation and chemical inactivation, thereby preserving bioactivity during systemic circulation [11]. From a drug delivery perspective, exosomes represent an endogenous nanoplatform with low immunogenicity and inherent targeting potential, which can be further engineered for precision therapy [10,11]. As shown in Figure 9, the combined delivery of Exo^EGCG^ and si-circRERE markedly improved therapeutic outcomes, highlighting the synergistic potential of co-encapsulating a small-molecule inhibitor and an RNA-based therapy within a unified exosomal system. Therefore, utilizing exosomes to simultaneously deliver si-circRERE and EGCG not only leverages the inherent benefits of nanodelivery—such as extended circulation time, targeted accumulation, and reduced off-target effects—but also establishes a promising combinatorial nanotherapeutic strategy for the treatment of AMI.

From a translational perspective, several key pathophysiological factors should be considered for future clinical application. First, the net effect on cardiomyocyte survival reflects the dynamic coexistence and crosstalk between pro-apoptotic and anti-apoptotic pathways rather than a single signaling cascade; the balance between Caspase-driven apoptosis and survival signals such as PI3K/Akt and Bcl-2 determines the ultimate fate of ischemic myocardium [2,3]. Second, cardiac responses undergo time-dependent transitions from acute protection to adaptive compensation and eventually maladaptive remodeling [2,3], meaning the efficacy of Exo^EGCG^ and circRERE inhibition may differ across the ischemic, reperfusion, and post-infarction repair phases. Third, the energetic state (mitochondrial function, ATP depletion) and microcirculatory status (capillary perfusion, no-reflow phenomenon) of ischemic/reperfused tissue directly affect exosome uptake, circRNA stability, and miRNA activity, which are critical for in vivo therapeutic outcomes [2,3,24].

Future investigations are warranted to explore whether the circRERE/miR-27a-3p/Caspase9 axis remains dysregulated in chronic myocardial remodeling and chronic heart failure following acute ischemic injury. Given the central role of sustained cardiomyocyte apoptosis in progressive ventricular dysfunction, it is worthwhile to investigate whether targeted inhibition of circRERE combined with Exo^EGCG^ administration holds potential for long-term intervention to limit pathological deterioration and preserve cardiac function during the chronic phase after myocardial infarction. In addition, engineered exosomes represent a novel and promising delivery platform for cardiovascular diseases, with excellent biocompatibility, low immunogenicity, and tunable cargo-loading capacity [10,11,12,13]. Therefore, further attempts can be made to apply Exo^EGCG^ in precise nucleic acid-based therapy.

There are certain limitations in the present study. First, HL-1 cells were used for in vitro experiments, which are immortalized cells rather than primary cardiomyocytes. Compared with primary cardiomyocytes, HL-1 cells may differ in metabolic characteristics and contractile function under hypoxic stress [25]. Future studies should validate our key findings using primary cardiomyocytes to enhance physiological relevance. Furthermore, the key regulatory factors governing circRERE biogenesis in AMI remain unclear. In addition, further systematic investigation is required to fully elucidate the precise mechanism by which Exo^EGCG^ modulates circRERE.

In conclusion, our study has identified the novel role of circRERE in myocardial ischemia, revealing its mechanism as a molecular sponge for miR-27a-3p to suppress apoptosis. Additionally, we delineated the signaling cascades and effector targets through which Exo^EGCG^ affords protection against AMI injury. The works provide innovative insights for the development of nanodrug delivery platforms and gene therapy strategies against AMI, and open up new avenues for the interventional treatment of cardiovascular diseases.

## 5. Conclusions

**Figure 10:**
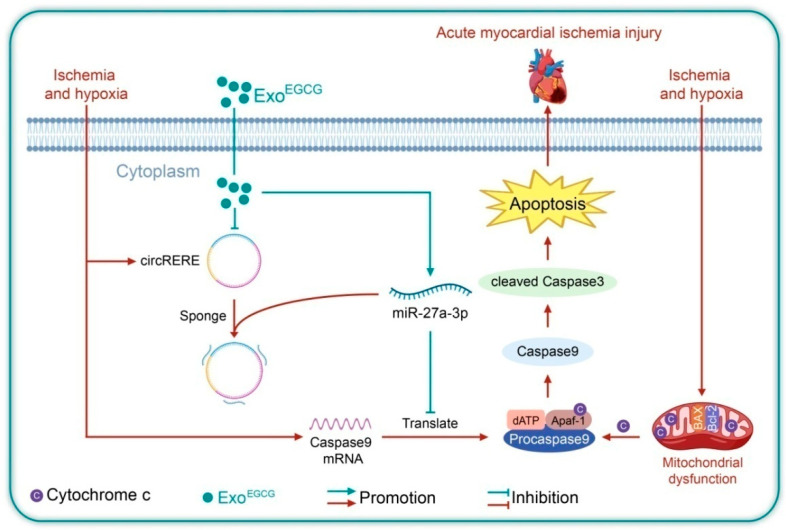
Exo^EGCG^ alleviated acute myocardial infarction injury via circRERE/miR-27a-3p/Caspase9 axis.

In summary, our findings highlight the circRERE/miR-27a-3p/Caspase9 signaling axis as a novel regulator in AMI pathogenesis, whose functional significance was further substantiated by the cardioprotective action of Exo^EGCG^ (Figure 10). These discoveries not only advance the mechanistic comprehension of AMI pathogenesis but also provide new targets for disease control.

## 6. Patents

Guilin Medical University, Application of circRERE inhibitors in the preparation of drugs for the treatment of ischemic heart disease, 202311251321.4 [P]. 18 June 2024.

## Data Availability

The original contributions presented in this study are included in the article/Supplementary Materials. Further inquiries can be directed to the corresponding author.

## Supplementary Materials

The following supporting information can be downloaded at: https://www.mdpi.com/article/10.3390/cells15090757/s1, Figure S1: Exo^EGCG^ exhibited dose-dependent cardioprotective effects but no obvious toxic effects; Figure S2: Exo^EGCG^attenuated AMI injury by inhibiting apoptosis; Figure S3: Silencing of miR-27a-3p partly reversed the effects of Exo^EGCG^; Figure S4: Overexpression of Caspase9 partly reversed the effects of Exo^EGCG^, while silencing of Caspase9 synergistically enhanced them; Table S1: Dose for in vitro experiments; Table S2: Dose for in vivo experiments; Table S3: Dose for in vivo experiments.

## Abbreviations

The following abbreviations are used in this manuscript:

AgoExpNum	the number of supported AGO CLIP-seq experiments
AMI	acute myocardial infarction
circExp	circRNA expression
EF	ejection fraction
EGCG	epigallocatechin gallate
Exo^EGCG^	exosomes derived from EGCG-treated hypoxic cardiomyocytes
Exo^Hypoxia^	exosomes derived from hypoxic cardiomyocytes
FS	shortened fraction
JncRto	junction ratio
MUT	mutant
RIP	RNA immunoprecipitation
SeqSml	sequence similarity
WT	wild-type
