# Supplementary material for: A Novel circRERE/miR-27a-3p/Caspase9 Signaling Axis Promotes Cardiomyocyte Apoptosis in Ischemic Myocardium: Insights from Epigallocatechin Gallate-Primed Exosomes
Source: Cells. 2026 Apr 23;15(9):757. doi: 10.3390/cells15090757 (PMC13162772; doi:10.3390/cells15090757)
Supplement: Supplementary file 1 [file cells-15-00757-s001.zip › cells-4246359-supplementary.pdf]

Supplementary Material

1 Supplementary Figures

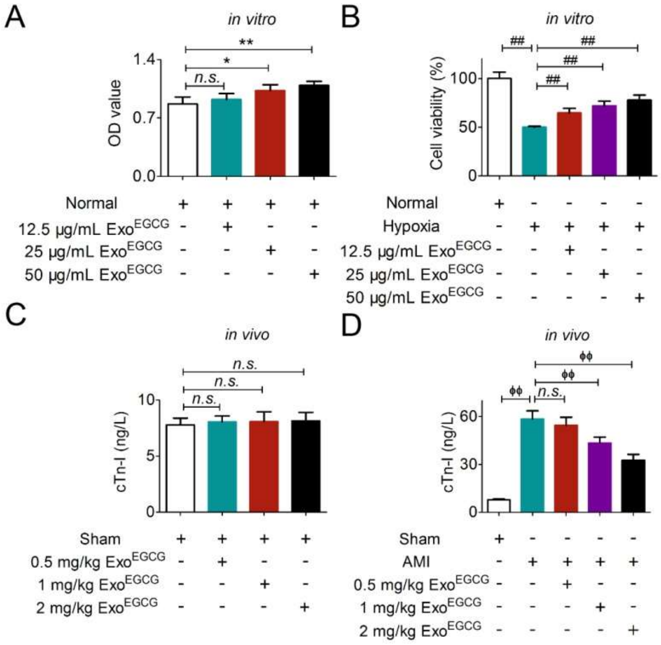

Figure S1. Exo^{EGCG} exhibited dose-dependent cardioprotective effects but no obvious toxic effects. Exo^{EGCG} exhibited no obvious toxic effects **(A)** in vitro and **(B)** in vivo. Exo^{EGCG} exhibited dose-dependent cardioprotective effects **(C)** in vitro and **(D)** in vivo. $n = 5$; $^{*}p$ <0.05, $^{**}p$ <0.01 vs. Normal; $^{\#\#}p$ <0.01 vs. Hypoxia; $^{\phi\phi}p$ <0.01 vs. AMI. +, treated; , not treated; n.s., no significance. Normal cardiomyocyte viability was considered as 100% viability.

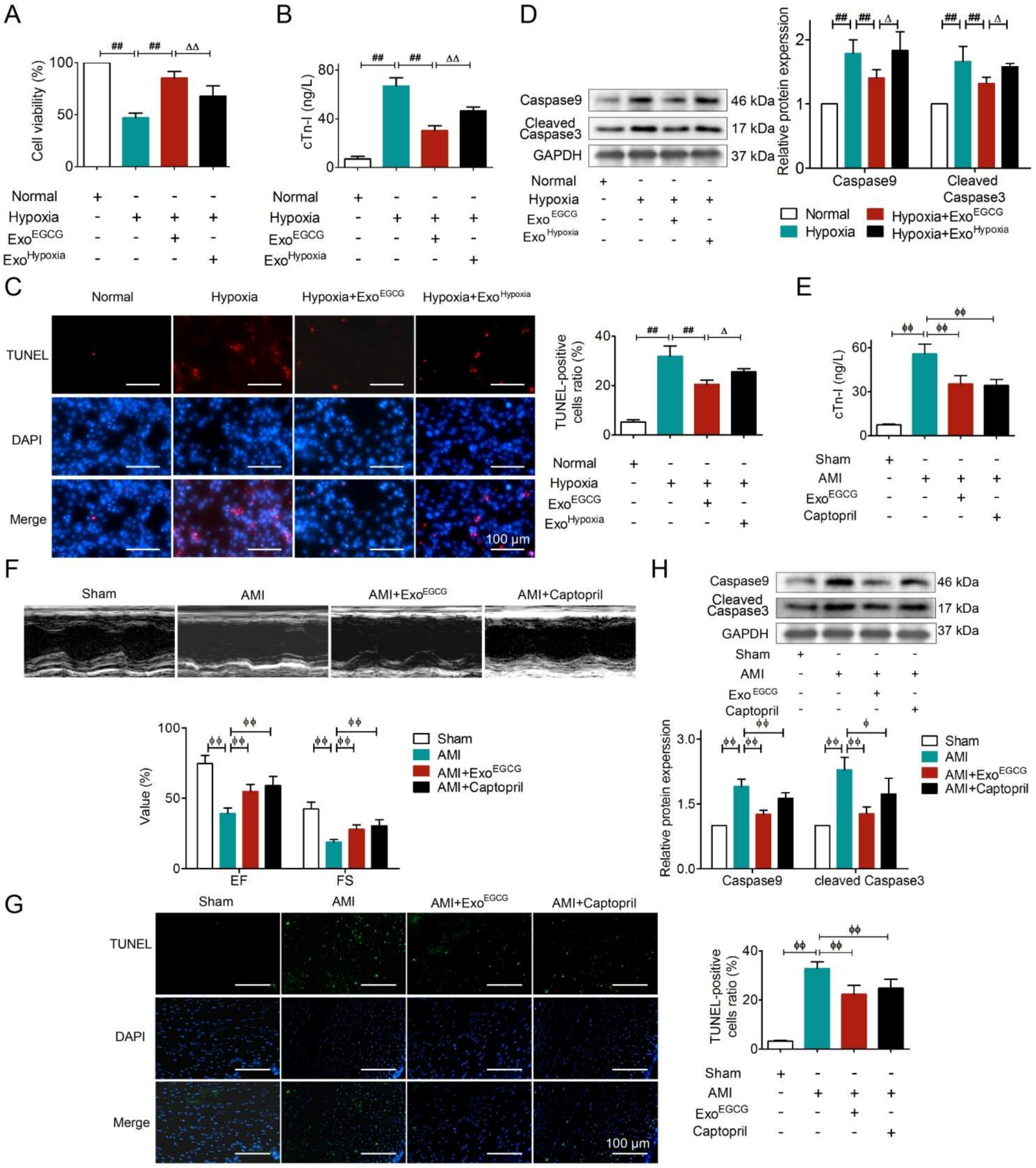

Figure S2. Exo^{EGCG} attenuated AMI injury by inhibiting apoptosis. Exo^{EGCG} **(A)** improved cell viability, **(B, E)** reduced cTn-I level, **(C, G)** lowered the apoptosis rate, in vitro $n = 5$, in vivo $n = 3$ **(D, H)** downregulated the protein levels of Caspase9 and Caspase3, **(F)** enhanced EF and FS. $n = 5$; $^{\#\#}p$ <0.01 vs. Hypoxia; $^{\Delta}p$ <0.05, $^{\Delta\Delta}p$ <0.01 vs. Hypoxia + Exo^{Hypoxia}; $^{\phi}p$ <0.05, $^{\phi\phi}p$ <0.01 vs. AMI. +, treated; -, not treated. Scale bars, 100 µm. Normal cardiomyocyte viability was considered as 100% viability.

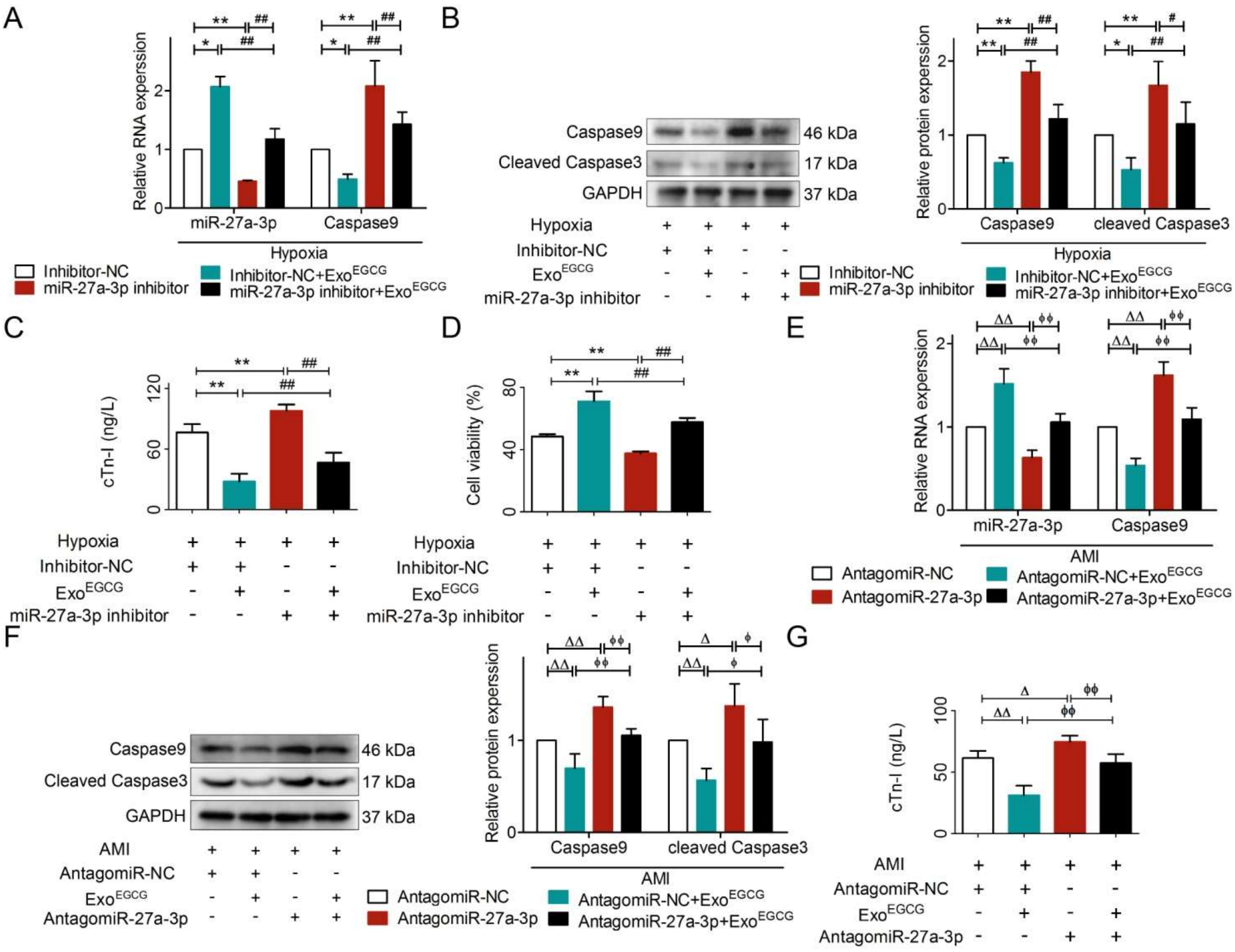

Figure S3. Silencing of miR-27a-3p partly reversed the effects of Exo^{EGCG}. In vitro and in vivo, silencing of miR-27a-3p partly reversed the effects of Exo^{EGCG} in mRNA levels of **(A, E)** miR-27a-3p and Caspase9, **(B, F)** protein levels of Caspase9 and cleave Caspase3, **(C, G)** cTn-I level, **(D)** cell viability, while Exo^{EGCG} also partially reversed above effects of silencing of miR-27a-3p. $n = 5$; $^{*}p$ <0.05, $^{**}p$ <0.01 vs. Hypoxia + Inhibibtor-NC; $^{\#}p$ <0.05, $^{\#\#}p$ <0.01 vs. Hypoxia + Exo^{EGCG} + miR-27a-3p inhibibtor; $^{\Delta}p$ <0.05, $^{\Delta\Delta}p$ <0.01 vs. AMI + Antagomir-NC; $^{\phi}p$ <0.05, $^{\phi\phi}p$ <0.01 vs. AMI + Exo^{EGCG} + AntagomiR-27a-3p. +, treated; -, not treated. Normal cardiomyocyte viability was considered as 100% viability.

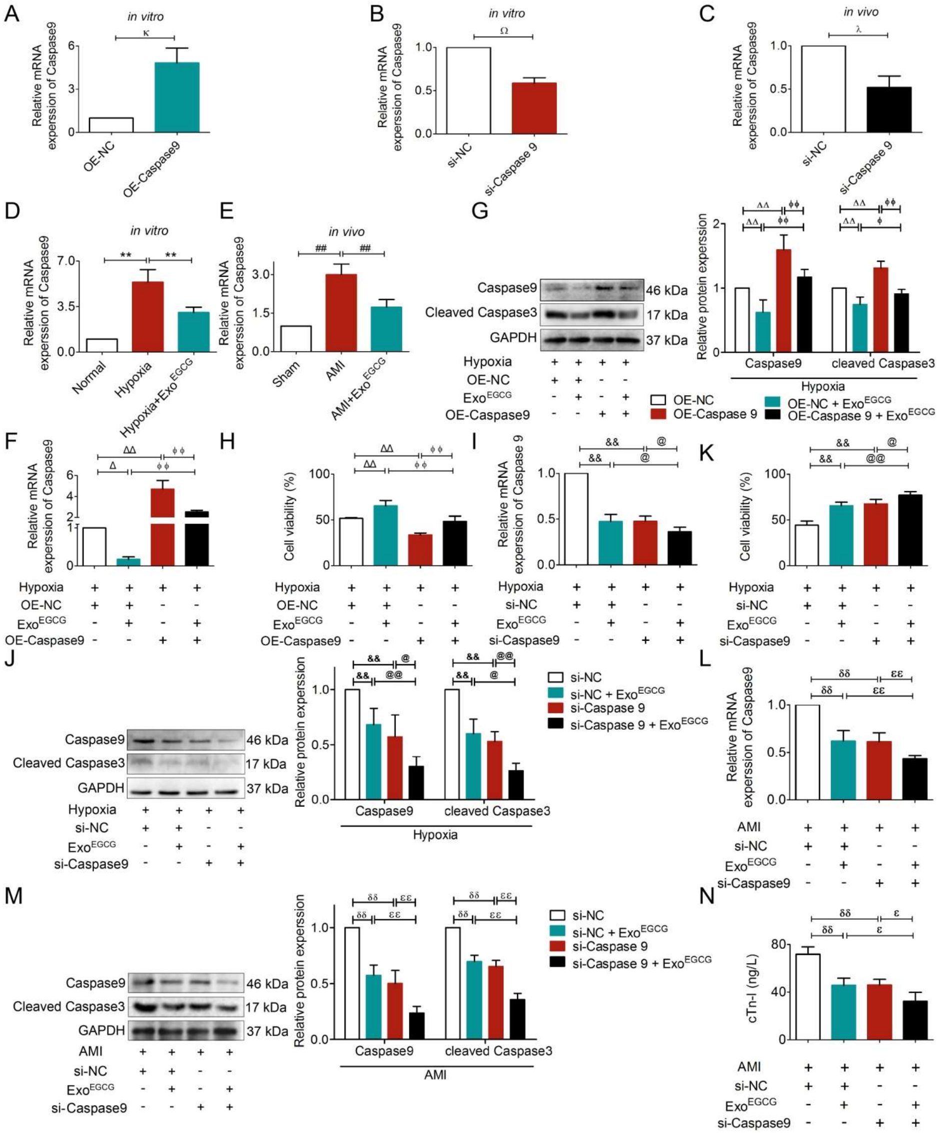

Figure S4. Overexpression of Caspase9 partly reversed the effects of Exo^{EGCG}, while silencing of Caspase9 synergistically enhanced them. Transfection effect of **(A)** OE-Caspase9 in vitro, **(B)** si-Caspase9 in vitro and **(C)** si-Caspase9 in vivo, $n = 3$. Caspase9 levels in AMI were regulated by Exo^{EGCG} **(D)** in vitro and **(E)** in vivo. Caspase9 overexpression partially reversed the effects of Exo^{EGCG} in **(F)** mRNA levels of Caspase9, **(G)** protein levels of Caspase9 and cleave Caspase3, **(H)** cell viability, while Exo^{EGCG} also partially reversed above effects of Caspase9 overexpression. In vitro and in vivo, silencing of Caspase9 synergistically enhanced the effects of Exo^{EGCG} in **(I, L)** mRNA levels of Caspase9, **(J, M)** protein levels of Caspase9 and cleave Caspase3, **(K)** cell viability, **(N)** cTn-I level, while co-administration of Exo^{EGCG} synergistically enhanced above effects of Caspase9 silencing. $n = 5$; $^{\kappa}p$ <0.05 vs. OE-NC; $^{\Omega}p$ <0.05 vs. si-NC in vitro; $^{\lambda}p$ <0.05 vs. si-NC in vivo; $^{**}p$ <0.01 vs. Hypoxia; $^{\#\#}p$ <0.01 vs. AMI; $^{\Delta}p$ <0.05, $^{\Delta\Delta}p$ <0.01 vs. Hypoxia + OE-NC; $^{\phi}p$ <0.05, $^{\phi\phi}p$ <0.01 vs. Hypoxia + Exo^{EGCG} + OE-Caspase9; $^{\&}p$ <0.05, $^{\&\&}p$ <0.01 vs. Hypoxia + si-NC; $^{@}p$ <0.05, $^{@@}p$ <0.01 vs. Hypoxia + Exo^{EGCG} + si-Caspase9; $^{\delta\delta}p$ <0.01 vs. AMI + si-NC; $^{\varepsilon}p$ <0.05, $^{\varepsilon\varepsilon}p$ <0.01 vs. AMI + Exo^{EGCG} + si-Caspase9. +, treated; -, not treated. Normal cardiomyocyte viability was considered as 100% viability.

2 Supplementary Tables

Table S1. Dose for in vitro experiments.

Medicine	Dosage
Exo^{EGCG} (optimal dose)	50 µg/mL
Exo^{EGCG} (low-dose group)	12.5 µg/mL
Exo^{EGCG} (middle-dose group)	25 µg/mL
Exo^{EGCG} (high-dose group)	50 µg/mL
$Exo^{Hypoxia}$	50 µg/mL

Table S2. Dose for in vivo experiments.

Medicine	Dosage	Route of administration
Exo^{EGCG} (optimal dose)	2 mg/kg	Stereotactic injection into myocardium
Exo^{EGCG} (low-dose group)	0.5 mg/kg	Stereotactic injection into myocardium
Exo^{EGCG} (middle-dose group)	1 mg/kg	Stereotactic injection into myocardium
Exo^{EGCG} (high-dose group)	2 mg/kg	Stereotactic injection into myocardium
Captopril	5 mg/kg	Caudal vein injection

Table S3. Dose for in vivo experiments.

Gene	Sequence
circRERE	Convergent primer
	Forward:5'-CTGAGACAAGGCGGCTGAATAGTAC-3'
	Reverse:5'-GACTCCAGGCATCCAGACAAGTTC-3'
	Divergent primer
	Forward:5'- AGGGATGTGTGATGGAGGCT-3'
	Reverse:5'-CTGGAAGTTTGGCCTGGTGG-3'
	Sjod primer
	Forward:5'-CTGAACACAGCGGCTGAATAG-3'
	Reverse:5'-GACTCCAGGCATCCAGACAAG-3'
miR-27a-3p	Forward:5'-AGTGGCTAAGTTCCGCAA-3'
Caspase 9	Forward:5'-CCTGCTTAGAGGACACAGGC-3'
	Reverse:5'-CGGGTCCAGCTTCACTACTC-3'
U6	Forward:5'-CGCTTCGGCAGCACATATAC-3'
	Reverse:5'-GGGCGTCATAGCCTTTCTTG-3'
GAPDH	Forward:5'-GACATGCCGCCTGGAGAAAC-3'
	Reverse:5'-AGCCCAGGATGCCCTTTAGT-3'
